# Subversion of allocation concealment in a randomised controlled trial: a historical case study

**DOI:** 10.1186/s13063-017-1946-z

**Published:** 2017-05-02

**Authors:** Andrew D. M. Kennedy, David J. Torgerson, Marion K. Campbell, Adrian M. Grant

**Affiliations:** 1 Health Services Research Unit, University of Aberdeen, Aberdeen, UK; 2 York Trials Unit, Department of Health Sciences, University of York, York, UK

**Keywords:** Randomisation, Sealed envelopes, Allocation concealment, Subversion of randomization

## Abstract

**Background:**

If the randomisation process within a trial is subverted, this can lead to selection bias that may invalidate the trial’s result. To avoid this problem, it is recommended that some form of concealment should be put into place. Despite ongoing anecdotal concerns about their susceptibility to subversion, a surprising number of trials (over 10%) still use sealed opaque envelopes as the randomisation method of choice. This is likely due in part to the paucity of empirical data quantifying the potential effects of subversion. In this study we report a historical before and after study that compares the use of the sealed envelope method with a more secure centralised telephone allocation approach in order to provide such empirical evidence of the effects of subversion.

**Methods:**

This was an opportunistic before and after study set within a multi-centre surgical trial, which involved 654 patients from 28 clinicians from 23 centres in the UK and Ireland. Two methods of randomly allocating subjects to alternative treatments were adopted: (a) a sealed envelope system administered locally, and (b) a centralised telephone system administered by the trial co-ordination centre. Key prognostic variables were compared between randomisation methods: (a) age at trial entry, a key prognostic factor in the study, and (b) the order in which ‘randomisation envelopes’ were matched to subjects.

**Results:**

The median age of patients allocated to the experimental group with the sealed envelope system, was significantly lower both overall (59 vs 63 years, *p* < 0.01) and in particular for three clinicians (57 vs 72, *p* < 0.01; 33 vs 69, *p* < 0.001; 47 vs 72, *p* = 0.03). No differences in median age were found between the allocation groups for the centralised system.

**Conclusions:**

Due to inadequate allocation concealment with the sealed envelope system, the randomisation process was corrupted for patients recruited from three clinicians. Centralised randomisation ensures that treatment allocation is not only secure but seen to be secure. Where this proves to be impossible, allocation should at least be performed by an independent third party. Unless it is an absolute requirement, the use of sealed envelopes should be discontinued forthwith.

## Background

It is widely accepted that randomised controlled trials are the method of choice for the evaluation of new clinical treatments. They differ from other prospective designs principally in the way in which the groups for comparison are generated. True random allocation leads to groups at trial entry that differ only by chance in potentially confounding prognostic factors, both known and unrecognised [1, 2].

Failure to secure random allocation can lead to selection bias that invalidates a trial’s results [3–6]. Knowledge of the next allocation may bias the decision to recruit a person or lead to participants with a better prognosis being assigned to one group rather than another [7]. Eliminating selection bias at trial entry requires two things: first, unpredictable sequencing of allocations, and second, secure allocation concealment, such that ‘irretrievable’ trial entry occurs before the assigned treatment is known [7].

If the allocation is deciphered this can lead to the possibility of subverting the randomisation, which introduces selection bias. Quantifying the frequency of subversion is difficult as it is a form of academic and clinical misconduct. Nevertheless, Berger [8] has collected over 30 examples where there was concern that the allocation schedule had been subverted and a survey of clinicians who have had experience of recruiting participants into clinical trials found that 16% of them kept a log of previous allocations in order to help them predict future ones [9]. To avoid the problem of subverting the allocations it is recommended that some form of concealment should be put into place to reduce the possibility of allocation subversion.

The use of sealed opaque envelopes is still often used in randomised trials, with a recent review finding that 11% of trials published in 2016 in four major medical journals used sealed envelopes [10]. This is despite Schulz [7] reporting, more than 20 years ago, instances where this approach can be manipulated by either opening envelopes in advance or through transillumination. A methodological study [11] that compared the significance levels of trials that mainly used sealed envelopes with those who used more secure, third-party, randomisation systems found that trials using less secure procedures had significantly smaller *p* values than those using a more secure allocation method. However, it was not possible in that review to identify individual instances of misallocation and the reporting of actual cases of misallocation is rare. In this present study we report a before and after study that compared the use of the sealed-envelope method with a more secure centralised telephone-allocation approach. Although this study took place in the mid-1990s we believe it still has relevance today, given the continuing prevalence of poorly concealed allocation in current clinical trials [10].

## Methods

The trial was a multi-centre randomised controlled trial in surgery in which age was judged to be a key prognostic variable. Age distribution was first described in an initial cohort of 327 participants randomised using a sealed envelope system [12], and then compared with a similar-sized cohort recruited after the introduction of a central telephone randomisation system.

### The sealed envelope system

The generation of the random sequence of envelope allocations was performed using a matched block method stratified by clinician. Allocation was assigned to each block using simple randomisation; this block sequence was then repeated swapping the order of the two treatments, giving an equal number of patients in the two treatment groups over the matched block. To ensure that the sequence could not be anticipated the block size was selected randomly to be 5, 10 or 15. The random sequence for these blocks was generated using a random number generator within the statistical analysis package SPSS, the seed calculated by multiplying the seconds and minutes portion of the computer’s internal clock. This generated a pseudo-random distribution in the range 0 to 1. Values <0.5 were allocated the control treatment, and those ≥0.5 to the experimental treatment.

These allocations were printed onto cards which were then sealed in sequentially numbered envelopes. This process was performed for each clinician on joining the collaborative trial group. At randomisation, the envelope number and patient identifying details were recorded on a form which was then returned to the trial administration centre to confirm recruitment.

### The centralised telephone randomisation system

The centralised system used a toll-free, dedicated telephone line. To recruit a patient the clinician now telephoned the trial administration centre giving details of the prognostic factors. Details were entered directly into a customised database package to generate the allocation. In addition to stratification by clinician, the allocation was minimised on five prognostic factors (age, sex, and three clinical factors - site of hernia, type of hernia and presence of recurrent hernia). These include age, which was split into three groups; the other factors were dichotomised.

### Statistical methods

For each randomisation system, the age distribution in the trial groups was compared first for all patients, and then for those recruited by the five clinicians who had recruited more than 25 people in the first time period; all other clinicians’ patients were combined to give a sixth group. Median ages were compared using the Mann-Whitney *U* test. Median differences in age between the groups (with 95% confidence intervals) were then generated. Apparent imbalances in the age distributions were further investigated by plotting the recruitment sequence against the envelope number, for those cases where this information was available.

## Results

**Fig. 1:**
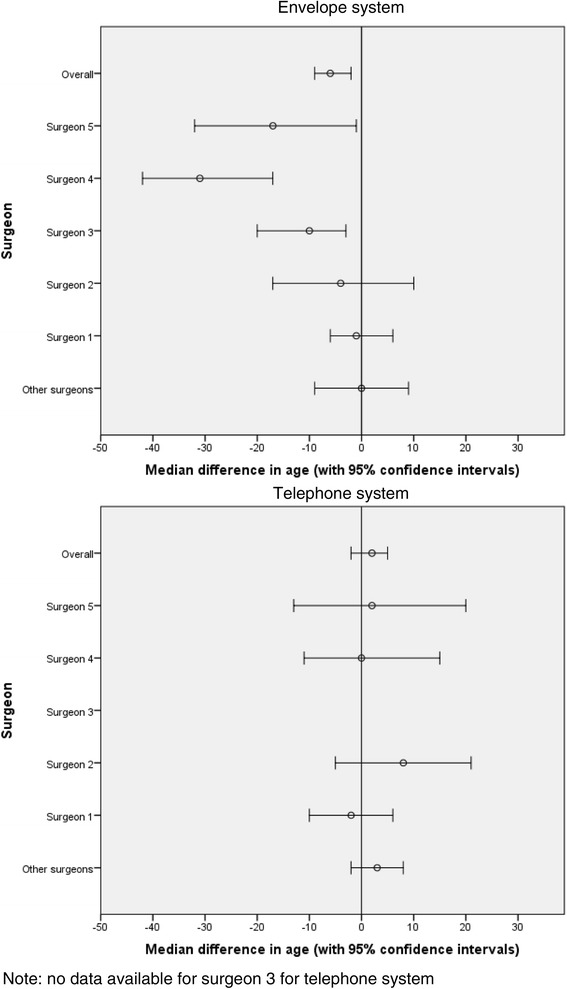
Comparison of the envelope system with the telephone system for median differences in age between the experimental and control groups

Table 1 summarises the age distributions first when the sealed envelope method was used and then when central telephone randomisation was employed. During the first time period the difference in the age distributions in the experimental and control groups is unlikely to reflect chance both overall (median age 59 vs 63 years, *p* < 0.01) and amongst those recruited by clinicians 3 (median age 57 vs 72 years, *p* < 0.01), 4 (median age 33 vs 69 years, *p* < 0.001), and 5 (median age 47 vs 72 years, *p* = 0.03). In contrast, differences in the period of central telephone randomisation were compatible with chance, random variation. Figure 1 shows the median differences in age with 95% confidence intervals, negative values indicating younger patients in the experimental group.

**Table 1:** Age distribution in trial groups: (a) using the sealed envelope method and (b) using central telephone randomization

	(a) Sealed envelope method							(b) Central telephone randomisation						
	Experimental			Control				Experimental			Control			
Clinician(s)	*n*	median	(IQR)	*n*	median	(IQR)	*p*	*n*	median	(IQR)	*n*	median	(IQR)	*p*
All	169	59	(40, 69)	158	63	(33, 63)	<0.01	162	59	(48, 69)	165	57	(44, 67)	0.37
1	64	62	(44, 70)	66	61	(44, 72)	0.84	38	57	(42, 66)	37	57	(50, 67)	0.62
2	19	43	(35, 65)	17	52	(43, 65)	0.60	13	60	(48, 71)	12	51	(45, 59)	0.24
3	24	57	(41, 67)	19	72	(53, 76)	<0.01	2	61	(-)	2	70	(-)	(-)
4	13	33	(25, 41)	13	69	(51, 74)	<0.001	14	63	(50, 70)	14	65	(41, 76)	0.99
5	14	47	(37, 65)	14	72	(53, 77)	0.03	12	57	(49, 71)	12	62	(34, 69)	0.91
Others	35	64	(45, 70)	29	59	(47, 71)	0.99	83	59	(48, 69)	88	56	(42, 67)	0.27

**Fig. 2:**
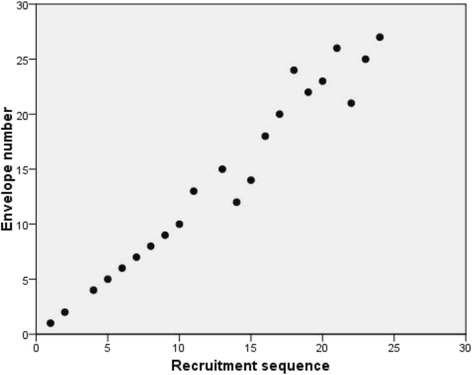
Plot of recruitment sequence against the envelope number for participants recruited by one surgeon

Plots of the recruitment sequence against the envelope number (Fig. 2) showed instances of skipping envelopes completely (for example, envelopes 3, 11 and 16 were not used at all), and evidence of envelopes being used out of sequence (for example patients numbered 15 to 20 were allocated using envelope numbers 14, 18, 20, 24, 22 and 23).

## Discussion

This study provides one of the very few empirical examples of subversion of randomisation and the consequences of such subversion for trial characteristics. In this study we detected the subversion problem by testing for age equivalence between the randomised groups. Although baseline testing is generally not promoted (e.g. Altman and Dore 1990 [4]) because in a properly randomised trial any differences in baseline are most likely due to chance, Berger [8] argues strongly that baseline testing still has a role in order to detect possible allocation subversion. He argues that because subversion is likely to be hidden from the investigators baseline testing is legitimate to reassure the investigators and readers that subversion is unlikely. The level of subversion is, however, required to be relatively substantial as in our example for it to effectively identify a subversion problem. A complementary approach, if trials use block randomisation, has been suggested to detect suspicious imbalances [13].

Although there is indirect and anecdotal evidence of important bias introduced by faulty randomisation procedures, investigators are understandably reluctant to describe such problems. The new surgical procedure that we were helping to evaluate was likely to need a longer period of general anaesthesia and so likely to be considered less suitable for older, frailer patients. We were therefore concerned at the apparent imbalance in age amongst the cohort recruited during the start-up phase of this trial; further investigations indicated that the system had been corrupted amongst patients recruited by three clinicians (their results were not used in the final trial analysis). The problem was restricted to the period when a sealed envelope method was used and was not seen after central telephone randomisation had been instituted.

The problem was one of inadequate concealment rather than with the generation of the sequence. There are a number of ways in which the allocation can be prematurely revealed before formal trial entry when sealed envelopes are used. More than one envelope can be opened until a desired allocation is found, the order in which patients are recruited may be manipulated such that a particular patient is allocated a chosen treatment, or the allocation may be discerned without opening the envelope (such as by transillumination) with the (biased) decision about trial entry dependent on the allocation. The plots of recruitment sequence against envelope number were used to explore these possibilities; these should show a straight line if the process is carried out correctly, although even this does not rule out biased entry. We did find examples of skipping envelopes and order changes amongst the participants recruited by the three clinicians. Order changes are unlikely to be an important factor; however, because in only two of the five times it occurred was the skipped allocation different from the card used.

Sequentially numbered sealed envelopes remain a widely used method for allocation in intended randomised trials, and occasionally are the only practical method of undertaking randomisation. For example, in a sample of ‘open’ trials published in major medical journals in 2016, about 11% were using sealed envelopes to conceal allocation [10] Clark et al. Our experience, however, which supports the previous anecdotal evidence reported by Berger [8] and Brown et al. [9] illustrates why the use of envelopes is more susceptible to corruption - often through well-intentioned, human ingenuity rather than other approaches - and why it is a less than ideal method of concealment. It also underlines the recommendation in the Consolidated Standards of Reporting Trials (CONSORT) statement on the reporting of randomised controlled trials that a full description be given of the actual methods used to generate and conceal trial allocations [14].

The mistake not to generate the allocation sequence from the executor of the treatment assigned is clearly illustrated. If a sealed envelope or other locally based method of random allocation has to be used a clear message from our study is that this should be the responsibility of an independent third party who has no direct involvement in the trial or in patient care. A centralised system such as the one in place for the main recruitment period of this trial is preferable.

In this trial the method of allocation was changed from blocked randomisation to minimisation, and allocation was minimised by centre. Using any form of restriction to the randomisation can, however, increase the risk of predictability and possible subversion through scheduling [13]. Indeed, a case study has been reported where subversion occurred despite using a central telephone randomisation service [15]. This was probably caused due to use of a predictable sequence of blocks in the randomisation.

## Conclusions

In summary, this study provides further empirical evidence that using sealed opaque sequentially numbered envelopes is an inadequate method of concealing treatment allocation and we recommend that, unless it is an absolute requirement, their use should be discontinued forthwith. Trialists should use other means to prevent allocation subversion.
